# The diagnostic dilemma for atypical presentation of progressive human Mpox

**DOI:** 10.1186/s12879-023-08852-2

**Published:** 2023-12-05

**Authors:** Syeda Sahra, Raul Orozco Villalobos, Brian M. Scott, Deidra J. Bowman, Joseph Sassine, Michelle Salvaggio, Douglas A. Drevets, Nelson Iván Agudelo Higuita

**Affiliations:** 1 Section of Infectious Diseases, Department of Internal Medicine, University of Oklahoma Health Sciences Center (OUHSC), Oklahoma City, OK 73104 USA; 2 Department of Internal Medcine, University of Oklahoma Health Sciences Center (OUHSC), Oklahoma City, OK 73104 USA

**Keywords:** Human Mpox, Persons living with HIV, Men who have sex with men, Atypical rash

## Abstract

**Background:**

Human mpox has increasingly been reported worldwide since May 2022, with higher incidence in men who have sex with men (MSM) and persons living with HIV (PLHIV) with presentation typical for generalized macules and papules.

**Case presentation:**

We are describing a case of human mpox, which presented as widespread, atypical round verrucous lesions that went undiagnosed in the community for six months and was treated with antibacterials and antifungals given the similarity to skin manifestations associated with endemic mycoses.

**Conclusions:**

Suspicion for human mpox should be high in young MSM and PLHIV who present with rash and mpox should be ruled out earlier.

## Background

Mpox is an infection endemic to West/Central Africa that came to public attention in the spring of 2022. The causative virus is a member of the same family as Smallpox (Variola virus) which belongs to the Orthopoxvirus genus. Although known in primates as far back as 1958, human transmission was not reported until 1970 in the Democratic Republic of Congo (DRC) [1]. In November 2022, the Centers for Disease Control and Prevention (CDC) and World Health Organization (WHO) made efforts to destigmatize the condition by changing the preferred terminology to "mpox." The symptoms include fever, headache, aches, chills, and, distinguishingly, lymphadenopathy with a rash that appears on the face and trunk and then spreads to the limbs. The rash follows five stages, from a macule to papule to vesicle and pustule, before scabbing and dissipating in seven to fourteen days [2]. The most common sites of skin lesions are the face, torso, extremities, genitals, scalp, palms of hands, soles of feet, mouth, and eyes [3]. The incidence of mpox continues to increase with more than 30,700 cases in US and 50 deaths with approximately 90,000 worldwide cases since the May 2022 outbreak from II-B Clade [4]. The recent outbreak has been noted to be in patients who are men who have sex with men, with a significant number of patients living with HIV [5]. Inoculation with the sister vaccine used for smallpox eradication also provides a degree of immunity for mpox [6]. The clinical presentations of mpox cases are often atypical when compared to previous outbreaks, sometimes without significant symptoms, and not classically centrifugal. Diagnosis is typically confirmed through PCR of the lesion or biopsy.

## Case presentation

A 27-year-old male with no known medical history had presented to his primary care physician in rural Texas, United States, complaining of a rash that acutely progressed to raised, circular lesions all over his body. These lesions started originally on his right forehead as a small papule two weeks prior but rapidly expanded to involve most of his body but the genitalia. Family history was not contributory. The patient had been using intravenous drugs (i.e., methamphetamines) for the last two years. He was a nonsmoker and did not drink alcohol. The patient had multiple unprotected sexual encounters with men in the past six years. He was born and raised in rural east Texas and had never traveled out of the state or the United States. He worked as a salesman in retail and cleaned the yard associated with house properties in his leisure time. No pets were reported at his house, but neighbors reportedly had chickens, pigs, and horses.

**Fig. 1:**
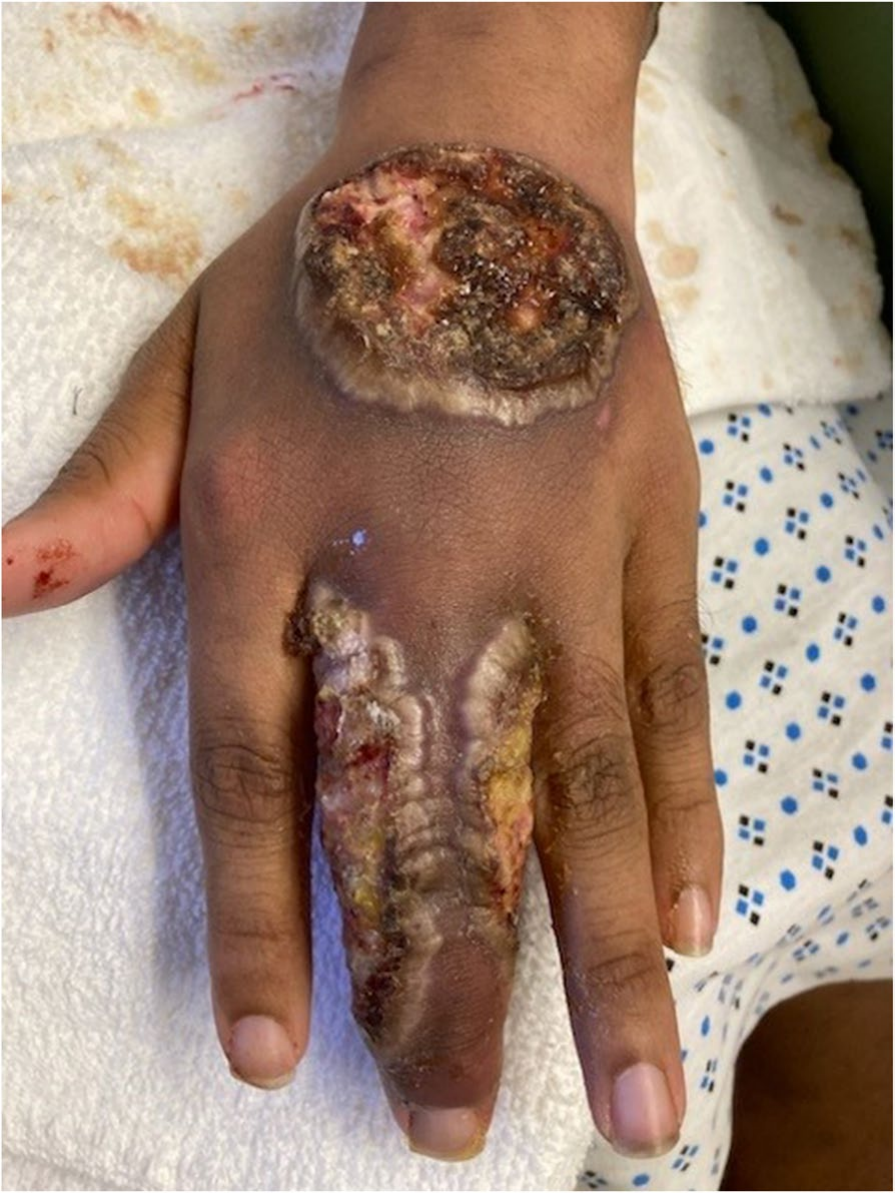
Raised circular lesion seen on dorsum of hand and third digit of left hand with elevated floor of ulcer, round margins and clear foul-smelling discharge noted on examination (Most representative image for this case)

**Fig. 2:**
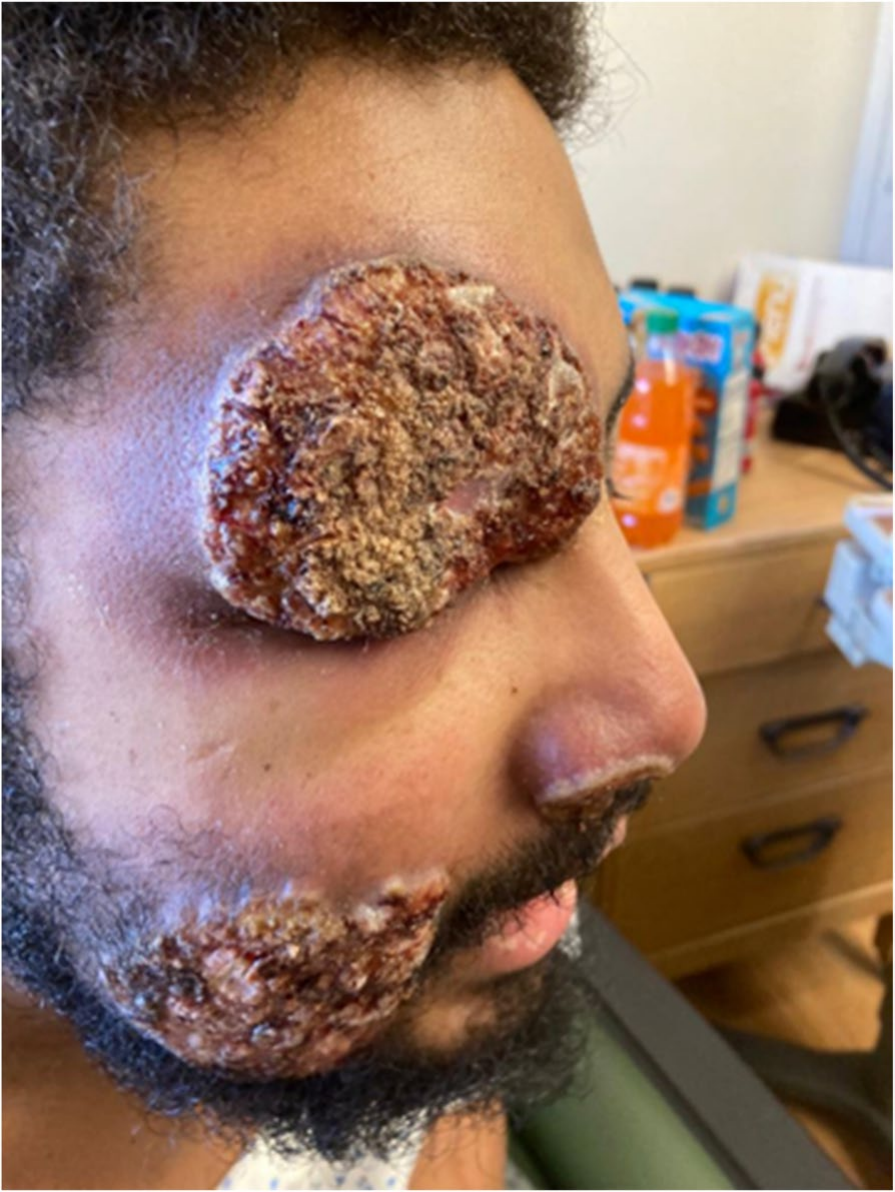
Raised circular lesion seen on left upper eyelid and cheek with elevated floor of ulcer, round margins and clear foul-smelling discharge noted on examination

**Fig. 3:**
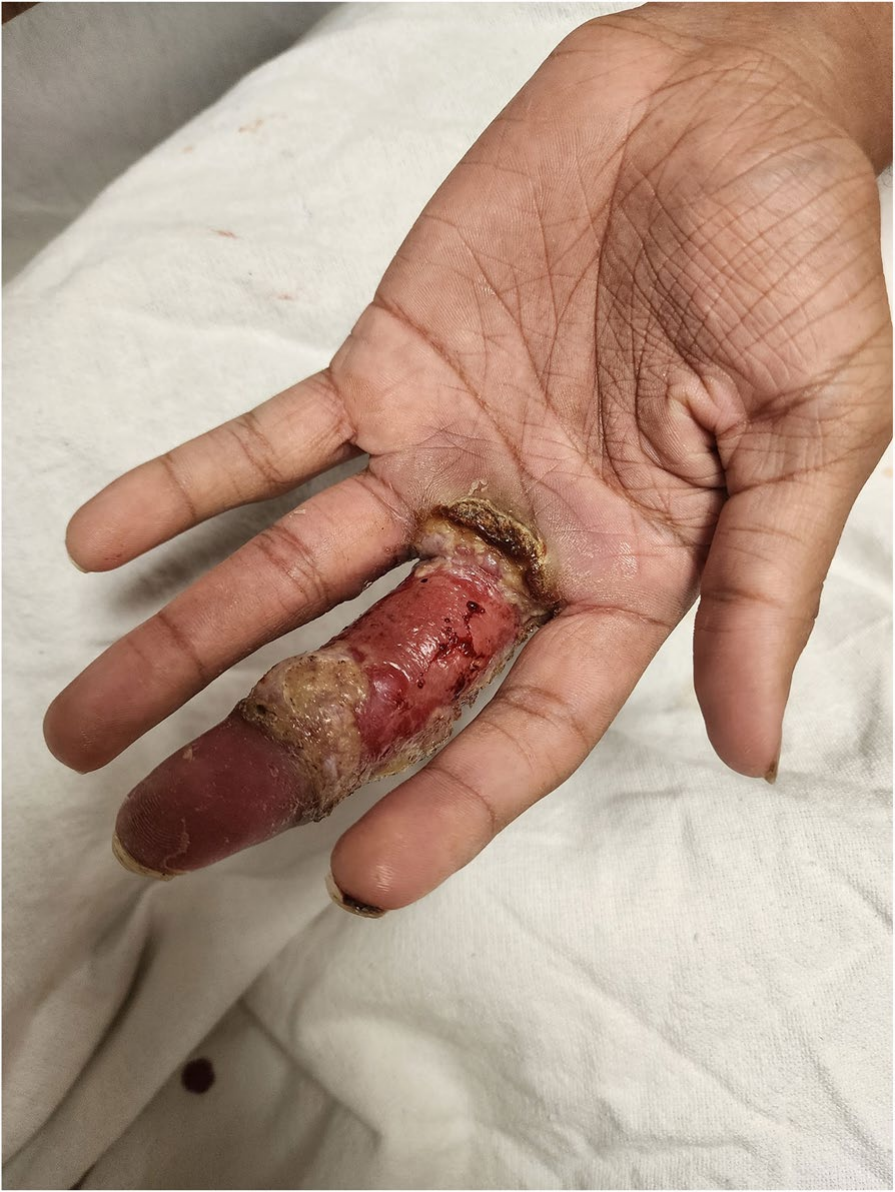
Raised circular lesion seen on palm of left hand involving third digit of left hand with complete ulceration of superficial dermis

The patient reported that the lesions started on his right forehead after direct trauma from a tree branch while cleaning the yard. The lesions were approximately 7 × 8 cm in diameter, had well-defined edges, were raised, and produced serous discharge (Figs. 1, 2, and 3). They had a foul-smelling discharge. The lesion over the right eye was nearly obscuring his visual field. The lesion on the left hand had extensive deroofing of the ulcer crater, and bone covered by a thin layer of musculature was visible.

**Fig. 4:**
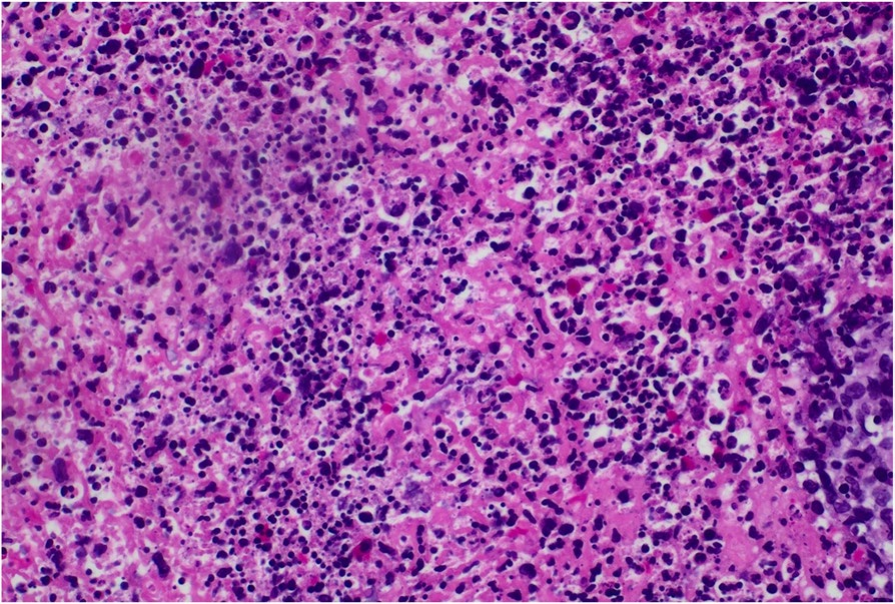
Cutaneous ulceration, dense neutrophilic infiltrate seen on skin biopsy taken from left hand. Site: Left hand. Stain: Hematoxylin & Eosin stain

The lesions had worsened despite short courses of oral antibiotics (levofloxacin, doxycycline, and linezolid) and progressively involved his right eyelid. Skin biopsies from multiple sites showed a dense neutrophilic infiltration (Fig. 4). The Gram stain, bacterial, fungal, and acid-fast bacilli (AFB) smear and cultures, and immunohistochemistry from the biopsies were unremarkable. The initial hematology and chemistry studies were within normal parameters. The patient was screened for sexually transmitted infections, and he tested positive for HIV (viral load of 100,000 copies/ml, CD4 count of 190 cells/mm3) and syphilis [rapid plasma regain (RPR) titer: 1:512]. The chest x-ray was unremarkable. He started antiretroviral therapy (bictegravir, emtricitabine & tenofovir alafenamide) and received three weekly intramuscular penicillin injections. The imaging of the left hand revealed underlying osteomyelitis in the second and third digits. Bacterial cultures of swabs from draining wounds from left hand and right shoulder isolated pan sensitive *Pseudomonas aeruginosa*, *Streptococcus dysgalactiae,* and *Staphylococcus aureus*. A Beta-D glucan, antigens and serology testing were obtained for *Sporothrix, Histoplasma, Blastomyces,* *Coccidiodes*, and *Paracoccidiodes* -all returned negative/non-reactive. Lateral flow antigen testing for *Cryptococcus* spp*.* was negative.

**Fig. 5:**
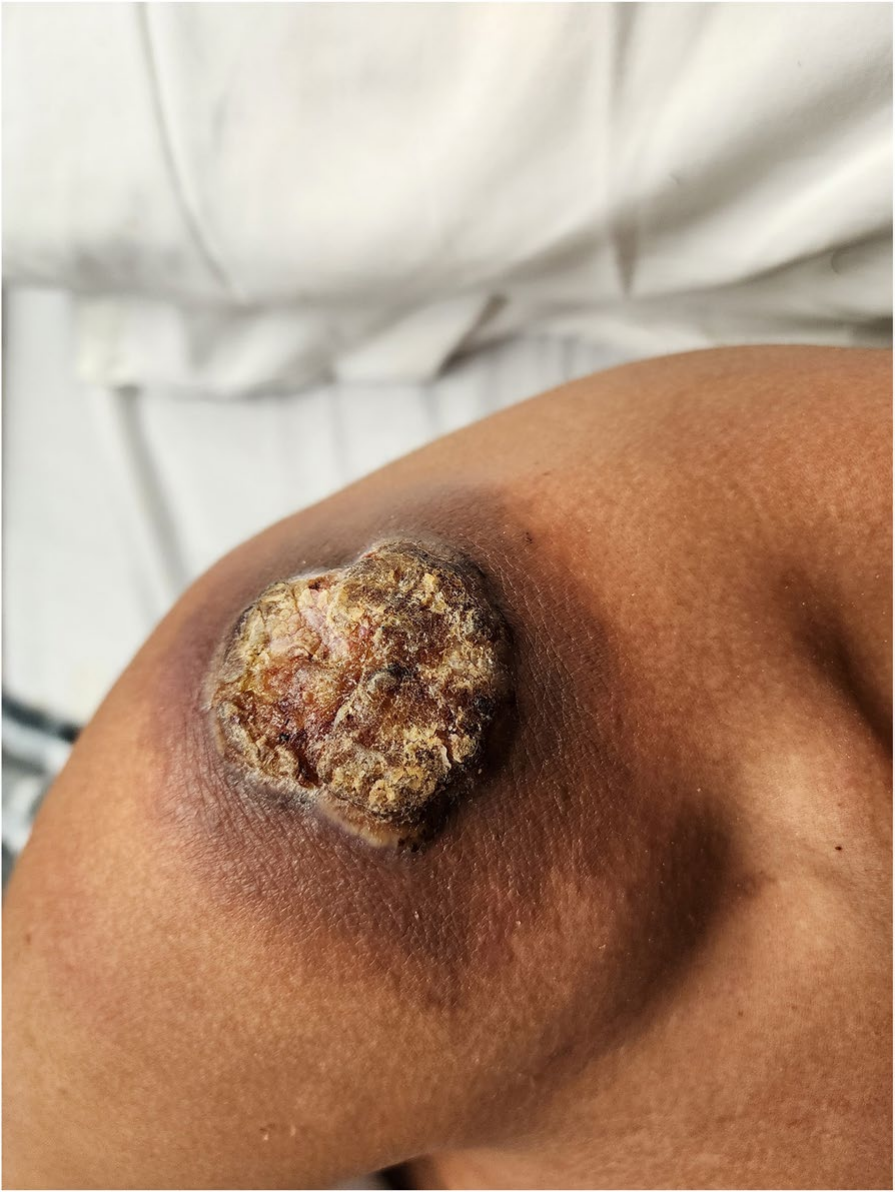
Worsening of skin lesion in terms of increased size and verrucae with dusking of neighboring margins seen after 2 weeks of intravenous amphotericin treatment

There was a high suspicion of systemic endemic mycoses, given the disseminated nature and appearance of the skin lesions and lack of response to courses of antibacterials given outpatient (levofloxacin, doxycycline, linezolid) and during hospitalization (cefepime and vancomycin). The patient was started on intravenous liposomal amphotericin B for two weeks, but no improvement was observed in the lesions (Fig. 5). The ophthalmological evaluation showed the presence of cotton wool spots bilaterally. No evidence of keratitis was seen. AFB staining, fungal staining, herpes simplex virus staining, bacterial, mycobacterial and deep fungal cultures of repeat punch biopsies taken from left hand and left ankle were negative. A presumptive diagnosis of endemic cutaneous mycosis was made, and the patient was discharged home on itraconazole 200 mg bid orally with outpatient follow up.

A metagenomic microbial plasma cell-free DNA (mcfDNA) next-generation sequencing assay (Karius) was sent, which revealed high levels of human mpox DNA (15468 DNA molecules per microliter).

**Fig. 6:**
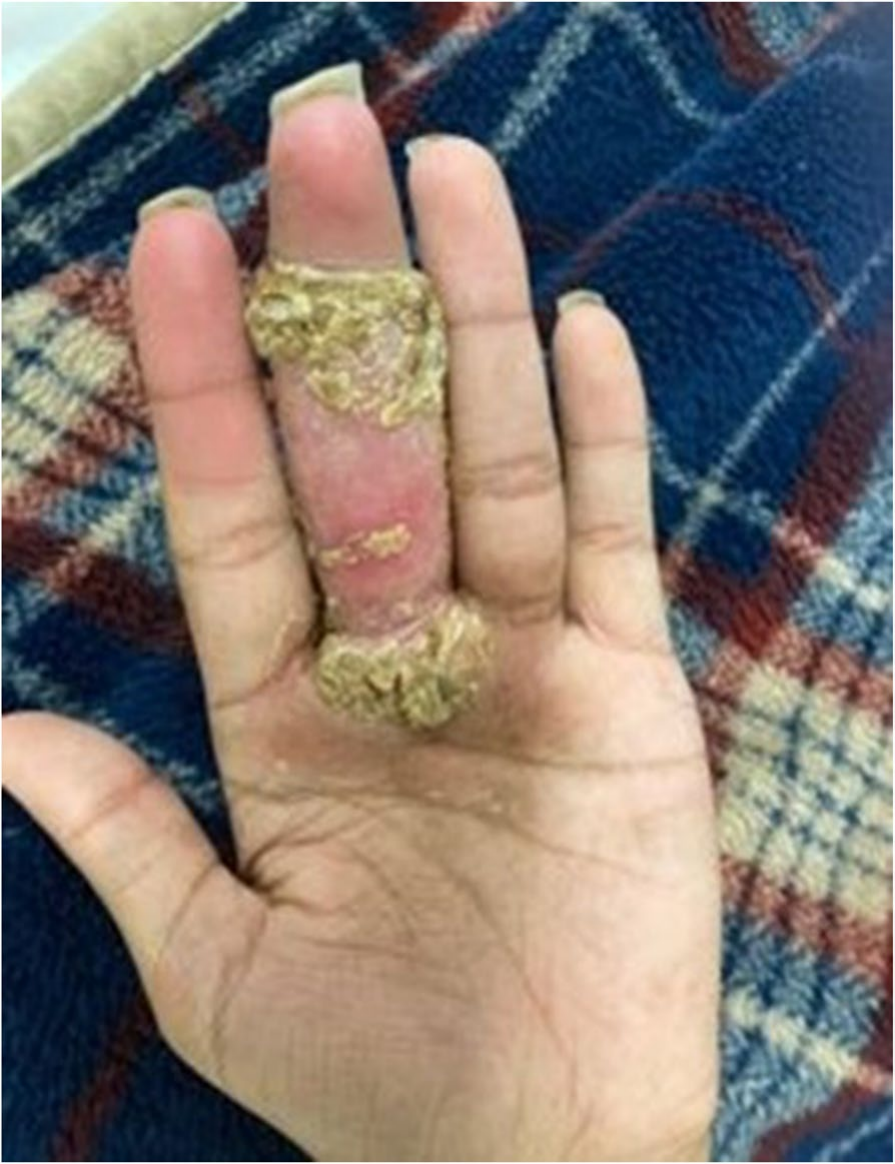
Re-epithelialization seen after day 5 of initiation of tecovirimat in left hand

**Fig. 7:**
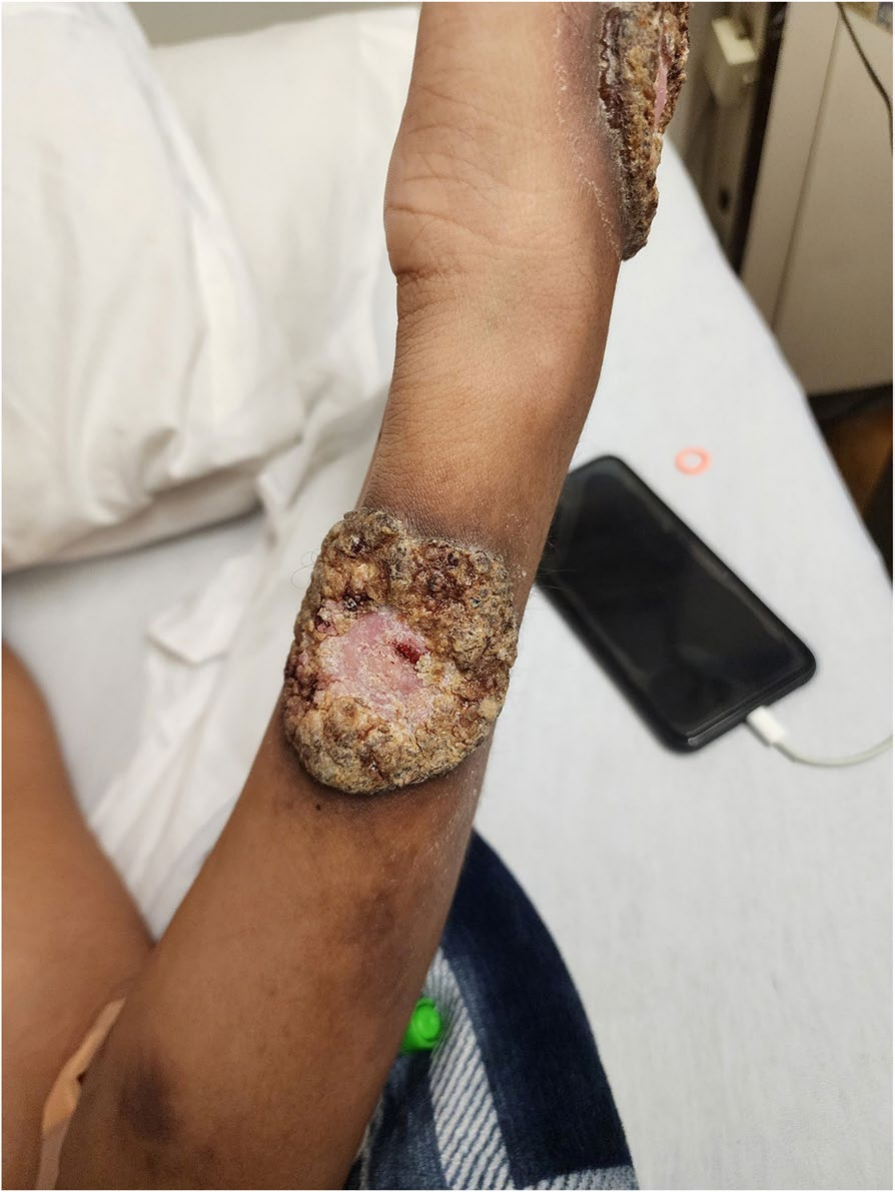
Re-epithelialization seen after day 5 of initiation of tecovirimat in left forearm

We contacted the patient once the Karius testing results were obtained and informed him of the diagnosis. The patient then recalled that his most recent sexual partner tested positive for human mpox four months ago and was treated successfully with tecovirimat. He had not brought this concern to the primary care physician or the infectious diseases team earlier as his lesions were remarkably different from his partner's lesions and other human mpox lesions he had searched online. The patient was also concerned about the partner’s privacy and potential stigma. Guidance from CDC via electronic consult services was obtained given the lesion’s very atypical appearance and extensiveness, including osteomyelitis and ophthalmological involvement. It was decided to start the patient on intravenous cidofovir and oral tecovirimat. He responded well with improvement in the lesions seen on 1 and 2-week follow-ups (Figs. 6 and 7).

## Discussion and conclusions

The differential diagnosis of widespread verrucous skin lesions in the Midwest and southern United States remains broad, including but not limited to disseminated *Sporothrix schenckii, *disseminated coccidioidomycosis, subacute progressive disseminated histoplasmosis, disseminated* b*lastomycosis, cutaneous leishmaniasis, ecthyma gangrenosum, verrucous pyoderma gangrenosum (PG) and inflammatory pseudotumor associated with herpes simplex infection. PG, an inflammatory neutrophilic dermatosis, is an important differential diagnosis of verrucous lesions, particularly in regions of endemic mycosis. It is manifested by crusted and painful ulcers with irregular heaped borders and nonspecific histopathological features including neutrophilic infiltration, granuloma formation, necrosis and fibrosis. PG responds well to systemic steroids, dapsone, clofazimine, while refractory cases have been managed by cyclosporin, azathioprine, and infliximab [7, 8]. Given the timing of appearance of lesions, histopathological findings and immediate respond to tecovirimat in our patient, our suspicion of verrucous PG lesions was low, and he was not trialed with immunosuppressive therapy.

The dermatological manifestations of human mpox continue to be updated as more cases with images are reported in the literature. Variations in presentation and clinical mimicry has led to unwarranted workups and treatments [9]. A study by Tarin-Vicente et al. (2022) reviewed 181 patients diagnosed with human mpox and reported clinical variability in genital, perianal, and oral lesions, with approximately 3 – 20 lesions in 80% of the sample size. The lesions were pustular in 90% of participants, vesicular in 26%, and papular in 21%. One-third of the patient population developed complications, including proctitis, tonsillitis, paraphimosis, and bacterial abscesses [10].

The key to timely diagnosis of these lesions in the geographical setting of endemic mycoses is to keep the risk factors in mind despite the atypical appearance of lesions. While the initial evaluation might be extensive given recent syphilis and the occupational exposure in an endemic location for fungal infections, any rash in a person living with HIV should raise concern for mpox. Co-infection of HIV, syphilis and human mpox has been reported in literature [11, 12]. The eruption of human mpox lesions can be a first opportunity for vulnerable populations to seek medical care and screening for all sexually transmitted infections including HIV, syphilis, chlamydia and gonorrhea should be performed in all cases of suspected or diagnosed mpox cases.

This case is unique because although most of the patient population of human mpox is immunocompromised owing to underlying HIV disease, the natural course of mpox lesions remains uniformly the same, where lesions start as macules and progress to papules and pustule before scabbing or scarring away in 2–3 weeks which did not happen in our patient. The relentless progression placed him at very high risk for progressive mpox, whose outcomes described in the literature have been dismal. A fatal case of progressive human mpox has been described with the mucocutaneous, pleuropulmonary, central nervous system, and gastrointestinal involvement where skin lesions were described to be > 2 cm ulcerated and purulent lesions on admission and later progression being strikingly similar to our patient with deep scarring rough indurated edges and elevated bed of the ulcer [13]. Most recently, another case of disseminated human mpox has been described in a young male with a similar course of illness, including syphilis and newly diagnosed HIV with viral load 150,565 copies/mL who, in addition to mpox lesions throughout his body had proctitis with PCR of skin swabs, brain, testicles being positive for mpox and bone marrow findings consistent with hemophagocytic lymphohistiocytosis. The patient did not have any improvement in his lesions despite multiple courses of tecovirimat and later succumbed secondary to multiorgan failure. Whole genome studies (WGS) were significant for high-level resistance to tecovirimat in vaccinia virus encoding the VP37 protein, which was later confirmed by phenotypic testing in 12 of 15 samples from viral cultures [14]. To the best of our knowledge and literature review, this is the first case of an anomalous presentation of mpox lesions where a favorable outcome was seen as manifested by images and one of the very few cases where the diagnosis was established using microbial cell free DNA (mcfDNA)testing due to atypical appearance leading to a meaningful difference in his longevity and quality of life.The role of mcfDNA is increasingly being recognized in infectious disease diagnostics. Most recently, Park et al. (2023) reported a case series of 12 patients with a direct correlation shown between mpox mcfDNA levels and mpox severity score [15]. An image-based deep convolutional neural network for pox (MPXV-CNN) has also been developed based on the dataset from 139,198 skin lesion images, including lesions in persons of color and involving different anatomical locations. MPXV-CNN has a promising potential for early diagnoses and decreasing transmission, which will be valuable in an outbreak setting in future [16].

Our patient responded extremely well to intravenous cidofovir and oral tecovirimat, reassuring that the multimodal approach has promising results even in cases where the lesions are advanced. Trifluridine drops have been used for ophthalmological involvement but were not used in this patient due to the absence of keratitis.

## Data Availability

Data sharing is not applicable to this article as no datasets were generated or analyzed during the current study. Microbiology reports, imaging and course of hospitalization are available as part of medical records. Corresponding author (Syeda Sahra, MD syeda-sahra@ouhsc.edu) can be contacted to request data.

## Abbreviations

AFB: Acid fast bacilli
CDC: Center for Disease Control
DNA: Deoxyribonucleic acid
HIV: Human Immunodeficiency Virus
PLHIV: Persons living with HIV
MSM: Men who have sex with men
WGS: Whole genome sequencing

## References

[1] Magnus Pv, Andersen EK, Petersen KB, Birch-Andersen A (1959). A pox-like disease in cynomolgus monkeys. Acta Pathologica Microbiologica Scandinavica.

[2] Jezek Z, Szczeniowski M, Paluku KM, Mutombo M (1987). Human monkeypox: clinical features of 282 patients. J Infect Dis.

[3] Ogoina D, Iroezindu M, James HI, Oladokun R, Yinka-Ogunleye A, Wakama P, Otike-Odibi B, Usman LM, Obazee E, Aruna O, Ihekweazu C (2020). Clinical course and outcome of human monkeypox in Nigeria. Clin Infect Dis..

[4] Centers for Disease Control and Prevention. Mpox: 2022 outbreak cases and data. 2023. https://www.cdc.gov/poxvirus/monkeypox/response/2022/index.html.

[5] Curran KG, Eberly K, Russell OO, Snyder RE, Phillips EK, Tang EC, Peters PJ, Sanchez MA, Hsu L, Cohen SE, Sey EK, Yin S, Foo C, Still W, Mangla A, Saafir-Callaway B, Barrineau-Vejjajiva L, Meza C, Burkhardt E, Smith ME, Murphy PA, Kelly NK, Spencer H, Tabidze I, Pacilli M, Swain CA, Bogucki K, DelBarba C, Rajulu DT, Dailey A, Ricaldi J, Mena LA, Daskalakis D, Bachmann LH, Brooks JT, Oster AM, Monkeypox, HIV, and STI Team (2022). HIV and sexually transmitted Infections among persons with monkeypox - eight U.S. jurisdictions, May 17-July 22, 2022. MMWR Morb Mortal Wkly Rep.

[6] Gessain A, Nakoune E, Yazdanpanah Y. Monkeypox. N Engl J Med. 2022;387(19):1783–93. Brooks JT, Marks P, Goldstein RH, Walensky RP. Intradermal Vaccination for Monkeypox - Benefits for Individual and Public Health. N Engl J Med. 2022;387(13):1151–1153. 10.1056/NEJMp2211311 36044621

[7] Oppermann K, Cocco AR, Heck R, Bonamigo RR (2018). PLECT or PPLECT? Granulomatous pyoderma gangrenosum in the differential diagnosis of the verrucous syndrome. An Bras Dermatol..

[8] Sachs D, Su L, Dlugosz A (2000). Verrucous annular ulcerated hip plaques. Diagnosis: superficial granulomatous pyoderma form of pyoderma gangrenosum. Arch Dermatol.

[9] Cowen EW, Tkaczyk ER, Norton SA, Leslie KS (2023). Mpox-A rapidly evolving disease. JAMA Dermatol.

[10] Tarín-Vicente EJ, Alemany A, Agud-Dios M, Ubals M, Suñer C, Antón A, Arando M, Arroyo-Andrés J, Calderón-Lozano L, Casañ C, Cabrera JM, Coll P, Descalzo V, Folgueira MD, García-Pérez JN, Gil-Cruz E, González-Rodríguez B, Gutiérrez-Collar C, Hernández-Rodríguez Á, de López-Roa P, Los Ángeles Meléndez M, Montero-Menárguez J, Muñoz-Gallego I, Palencia-Pérez SI, Paredes R, Pérez-Rivilla A, Piñana M, Prat N, Ramirez A, Rivero Á, Rubio-Muñiz CA, Vall M, Acosta-Velásquez KS, Wang A, Galván-Casas C, Marks M, Ortiz-Romero PL, Mitjà O (2022). Clinical presentation and virological assessment of confirmed human monkeypox virus cases in Spain: a prospective observational cohort study. Lancet.

[11] Ordoñez-González I, López-Zamora B, Medina G, Reyes-Navarro GV, Navarro AO, Cruz-Domínguez MP, Vera-Lastra O, Saavedra MÁ (2022). Human monkeypox coinfection with syphilis in an immunocompromised patient. Dermatol Rep.

[12] de Sousa D, Patrocínio J, Frade J, Correia C, Borges-Costa J, Filipe P (2022). Human monkeypox coinfection with acute HIV: an exuberant presentation. Int J STD AIDS.

[13] Caria J, Vara-Luiz F, Maia I, Joosten A, Val-Flores L, Pinheiro H, Póvoas D, Germano N, Maltez F (2023). Fatal case of progressive mpox in a patient with AIDS-Viral enteropathy and malabsorption demanding the use of full parenteral ARV and endovenous cidofovir. Infect Dis Rep.

[14] Alarcón J, Kim M, Terashita D, Davar K, Garrigues JM, Guccione JP, Evans MG, Hemarajata P, Wald-Dickler N, Holtom P, Garcia Tome R, Anyanwu L, Shah NK, Miller M, Smith T, Matheny A, Davidson W, Hutson CL, Lucas J, Ukpo OC, Green NM, Balter SE (2023). An mpox-related death in the United States. N Engl J Med.

[15] Park SY, Lindner MS, Brick K, Noll N, Ounit R, Noa LJ, Sabzwari R, Trible R, Sniffen JC, Roth P, Khan A, Rodriguez A, Sahra S, Davis MJ, Brar IS, Balasundaram G, Nolte FS, Blauwkamp TA, Perkins BA, Bercovici S (2023). Detection of Mpox virus using microbial cell-free DNA: the potential of pathogen-agnostic sequencing for rapid identification of emerging pathogens. J Infect Dis.

[16] Thieme AH, Zheng Y, Machiraju G, Sadee C, Mittermaier M, Gertler M, Salinas JL, Srinivasan K, Gyawali P, Carrillo-Perez F, Capodici A, Uhlig M, Habenicht D, Löser A, Kohler M, Schuessler M, Kaul D, Gollrad J, Ma J, Lippert C, Billick K, Bogoch I, Hernandez-Boussard T, Geldsetzer P, Gevaert O (2023). A deep-learning algorithm to classify skin lesions from mpox virus infection. Nat Med.

